# Development of a machine learning-based risk prediction model for early-stage pneumoconiosis: a retrospective study

**DOI:** 10.3389/fmed.2025.1730472

**Published:** 2026-01-07

**Authors:** Xin Jin, Xinghua Li, Zhaobo Guan, Hao Xu, Shaojie Li, Yaru Jiang, Lin Zhao, Wanping Wang, Zhenyu Li

**Affiliations:** 1 Changzhi People’s Hospital Affiliated to Shanxi Medical University, Changzhi, China; 2 Modern Research Center for Traditional Chinese Medicine of Shanxi University, Taiyuan, China; 3 The Key Laboratory of Effective Substances Research and Utilization in TCM of Shanxi Province, Taiyuan, China; 4 Key Laboratory of Chemical Biology and Molecular Engineering of Ministry of Education, Shanxi University, Taiyuan, China

**Keywords:** pneumoconiosis, LASSO regression, logistic regression, SVM model, SHAP analysis

## Abstract

**Background:**

The diagnosis of occupational pneumoconiosis requires more accurate predictive models. The purpose of this study is to screen blood markers associated with early pneumoconiosis development from blood routine indicators in physical examination data, and to develop a highly sensitive and accurate clinical prediction model using machine learning (ML) algorithms to promote early diagnosis and timely intervention.

**Method:**

Data on age and various blood test results were collected from the results of the physical examination. Predictors were analyzed using the Least Absolute Contraction and Choice Operator (LASSO) and multiple logistic regression. A total of 9 ML models were evaluated in this study, including Logistic Regression (LR), eXtreme Gradient Boosting (XGBoost), Light Gradient Boosting Machine (LightGBM), Random Forest (RF), Adaptive Boosting (AdBoost), Gaussian Naïve Bayes (GNB), Multilayer Perceptron (MLP), Support Vector Machine (SVM), and K-Nearest Neighbors (KNN). We compared the performance of the models based on the following criteria: ROC, accuracy, sensitivity, specificity, positive predictive value (PPV), negative predictive value (NPV), F1, the decision curve analysis (DCA), calibration curves, and precision-recall (PR) curves of the 9 models. Shapley Additive exPlanations (SHAP) interpretations are developed for personalized risk assessment.

**Results:**

In this study, 6 risk variables associated with the development of pneumoconiosis were identified, including White Blood Cell (WBC), Platelet Distribution Width (PDW), Total Bilirubin (TB), Absolute Neutrophil Count (ANC), Alanine Aminotransferase (ALT) and Aspartate Aminotransferase (AST). SVM was considered the optimal model and showed a good clinical applicability evaluation. SHAP analysis was employed to define the contributions of 6 variables to the progression of pneumoconiosis.

**Conclusion:**

The indicators ultimately established as being associated with pneumoconiosis progression were WBC, PDW, TB, ANC, ALT and AST. The ML algorithm combined blood biochemical indicators to determine the risk factors associated with the occurrence of pneumoconiosis. The SVM model performs well and has the potential to improve early detection and diagnosis in clinical practice.

## 1 Introduction

Pneumoconioses are defined as a spectrum of interstitial lung diseases caused by the alveolar deposition of inhaled respirable particles, typically under 5 μm in diameter (1). The disease manifests pathologically as chronic pulmonary inflammation and fibrosis (2, 3). Chronic pulmonary inflammation serves as a critical driver of fibrotic progression, ultimately serving as a key pathogenic precursor to pneumoconiosis (4). Pneumoconiosis includes silicosis, siliconosis, mixed pneumoconiosis, coal workers’ pneumoconiosis, asbestosis, chronic beryllium disease, alumina, hard metal pneumoconiosis, and some less severe pneumoconiosis (3, 5).

Pneumoconiosis remains a globally prevalent disease, with a persistently high incidence in recent years. According to 2023 World Health Organization estimates, approximately 15,000 to 20,000 new cases of pneumoconiosis are reported annually worldwide (6). The majority of pneumoconiosis cases are concentrated in developing countries. Of these, China reports the highest number of cases globally, more than half of the world’s total, which is largely attributable to its large-scale coal mining sector (7). The annual incidence of pneumoconiosis associated with coal mining and construction in India has risen by roughly 3,000 cases per year. Concurrently, an increasing trend is observed in developed countries such as the United States and Australia, despite their well-established healthcare infrastructures, rigorous safety protocols, and extensive mechanization in mining (8). The persistent status of pneumoconiosis as a major global health issue is attributable to its progressive and irreversible nature. This underscores the necessity of early detection and preventive measures for at-risk populations, particularly those exposed to occupational dusts such as coal (9).

Consequently, significant emphasis has been placed on the prevention and early diagnosis of pneumoconiosis in China. Compulsory annual medical surveillance, encompassing chest X-rays and hematological parameters, is mandated for high-risk occupational groups such as coal miners, construction workers, and stone processing operatives. Currently, the diagnostic gold standard for coal workers’ pneumoconiosis (CP) remains defined by a confirmed history of occupational dust exposure coupled with characteristic imaging abnormalities (6). A notable limitation of imaging studies is their inability to reliably assess lung function in CP, as pulmonary function can vary substantially among patients with similar radiographic presentations. This discrepancy hinders accurate severity assessment and complicates clinical management. Patients with early-stage pneumoconiosis are often clinically silent and may present with no obvious abnormalities on chest X-rays (10). Moreover, follow-up imaging after a physical examination also incurs high diagnostic costs. Therefore, it may play a small role in the early diagnosis of pneumoconiosis.

Elevated levels of serum biomarkers, including interleukin-6, interleukin-8, para-hydroxyphenylpropylene, and transforming growth factor-β1, have been reported in patients with pneumoconiosis, supporting their potential role in facilitating early diagnosis (11, 12). Given that pneumoconiosis involves early pulmonary inflammation, the neutrophil–platelet ratio (NPR) has emerged as a promising inflammatory marker, with demonstrated associations across a spectrum of disease outcomes (13). Studies have demonstrated that the pneumoconiosis group exhibits a significantly lower lymphocyte-to-monocyte ratio (LMR) and a higher neutrophil-to-lymphocyte ratio (NLR) compared to the control group (*p* < 0.05) (14). Despite being routine assessment items, the diagnostic value of an expanded spectrum of serum markers for the screening and early detection of pneumoconiosis is not yet established, representing a significant gap in the current literature.

The application of machine learning (ML) for big data analytics presents a substantial promise, owing to its considerable capacity for processing and interpreting large volumes of complex healthcare data (15). ML offers distinct advantages over traditional biostatistical methods, including enhanced flexibility and scalability, enabling applications in risk stratification, diagnostic classification, and survival prediction. Furthermore, these algorithms are capable of integrating multimodal data—such as demographic information, laboratory results, imaging features, and clinical narratives—to improve predictions of disease risk, diagnosis, prognosis, and optimal treatment strategies (16, 17). Thus, the application of ML algorithms to large-scale patient datasets enables the identification of latent correlations and complex patterns, which can assist healthcare professionals in predicting disease onset with greater accuracy (18).

In the past decade, multiple ML algorithms have been utilized in pneumoconiosis-related disease prediction and diagnosis. Yankun Ma and colleagues integrated CT imaging with occupational health surveillance data to develop a cross-modal model capable of identifying the preclinical stage of CWP. This approach enables the timely recognition and intervention of at-risk miners, potentially slowing disease progression and improving long-term outcomes (19). Chang Liu et al. collected chest radiographs from all enrolled subjects and employed a deep learning–based U-Net architecture to extract lung fields from pneumoconiosis X-ray images. By allowing the neural network to learn discriminative radiographic features, their method achieved accurate staging of pneumoconiosis and further advanced the application of deep learning in the diagnosis and screening of the disease (20). Xiaobing Li and co-workers focused on patients with chronic fibrotic lung disease and developed an interpretable multimodal machine-learning framework that integrates CT radiomics with clinical parameters to predict the progression of pulmonary fibrosis. Their methodological strategy is broadly extendable to other occupational and interstitial lung diseases, highlighting its wider applicability within respiratory medicine (21). Yafeng Liu and colleagues aimed to analyze whole-lung radiographic texture patterns from a large set of chest CT scans and to integrate these features with multiple machine-learning classifiers to construct radiologic markers for identifying individuals at high risk of pneumoconiosis. The overarching goal of their work was to support radiologists in rapidly recognizing abnormal chest imaging findings and to assist clinicians in providing timely intervention for susceptible, high-risk workers, thereby contributing to the prevention and reduction of pneumoconiosis incidence (22). The study conducted by Blanca Priego-Torres and co-workers included chest radiographs obtained over multiple years from individuals engaged in polishing, cutting, or surface finishing of engineered stone. Their work introduced a deep-learning–based algorithm for the preprocessing of X-ray images and evaluated the full analytical pipeline for its potential to automatically screen and stage silicosis (23). This comparative approach has enabled the screening of ideal biomarkers with robust sensitivity and specificity, facilitating the transition of these indicators from secondary prevention to the clinical diagnosis of CP (24). The study by Sun et al. aimed to screen for predictive biomarkers from routine blood parameters, with the objective of developing a comprehensive, multi-laboratory prediction model for silicosis by employing ML methods (25). Notably, however, these studies have not employed large clinical cohorts, and a predictive model utilizing routine blood biomarkers to provide individualized risk stratification for early pneumoconiosis in at-risk, undiagnosed workers remains unaddressed. Therefore, this study aimed to employ ML to compare hematological profiles between pneumoconiosis patients and individuals with occupational coal dust exposure, and to develop a clinical prediction model with high sensitivity and accuracy. Based on readily accessible and cost-effective routine blood parameters, the model aims to identify risk factors for early-stage pneumoconiosis progression and predict individual disease outcomes. Ultimately, it could enable coal miners to assess their personal risk of developing pneumoconiosis using data from routine health examinations, thereby facilitating effective intervention. Furthermore, this efficient predictive tool may assist healthcare providers in identifying high-risk populations and achieving early and rapid screening and diagnosis. The findings are expected to provide a theoretical foundation for personalized prevention and treatment strategies.

## 2 Materials and methods

### 2.1 Study design and participants

This retrospective study enrolled male patients who underwent medical examinations at the Department of Occupational Disease Prevention and Control of a tertiary hospital in Shanxi Province, China, between 2022 and 2024. In China, national occupational protection policies strictly prohibit female workers from engaging in underground coal mining operations to mitigate occupational hazards and protect reproductive health. Consequently, the coal mining workforce at our study site consists exclusively of male workers, and no female patients were available for inclusion in the study. Inclusion criteria are as follows: (1) patients diagnosed with stage I coal workers’ pneumoconiosis (CWP) according to China’s National Occupational Health Standard *Diagnosis of Occupational Pneumoconiosis* (GBZ 70–2015) (26); (2) healthy coal miners who had been exposed to coal dust but had not developed pneumoconiosis; (3) patients for whom complete hematological data were available. A single measurement for each biomarker was obtained from routine annual health examinations conducted by the mining company. Historical laboratory data, repeated tests, and outlier values were not included in the analysis. Exclusion criteria are as follows: (1) patients diagnosed with pneumoconiosis were excluded if they presented with severe comorbidities, including but not limited to lung cancer, cor pulmonale, respiratory failure, spontaneous pneumothorax, pulmonary tuberculosis, severe infectious diseases, or pulmonary hypertension; (2) patients with missing hematological data were excluded. This study has been approved by the Medical Ethics Committee of Changzhi People’s Hospital (Approval No.: 2025K043).

### 2.2 Grouping methods and study indicators

All participants were divided into two groups: those diagnosed with coal workers’ pneumoconiosis (CP) group and healthy coal miners who were exposed to coal dust but without pneumoconiosis (Control group). A total of 22 demographic and blood test indicators were collected from all patients, including: Age, Red Blood Cell (RBC), Platelet (PL), Hemoglobin (HB), Absolute Neutrophil Count (ANC), Neutrophil Percentage (NP), Absolute Lymphocyte Count (ALC), Lymphocyte Percentage (LP), Absolute Eosinophil Count (AEC), Eosinophil Percentage (EP), Absolute Basophil Count (ABC), Basophil Percentage (BP), Platelet Distribution Width (PDW), Alanine Aminotransferase (ALT), Aspartate Aminotransferase (AST), White Blood Cell (WBC), Mean Platelet Volume (MPV), Total Cholesterol (TC), Triglycerides (TR), High Density Lipoprotein Cholesterol (HDL-C), Low Density Lipoprotein Cholesterol (LDL-C), Total Bilirubin (TB). Prior to statistical analysis, the data were screened for outliers and missing values. No outliers were identified, and all variables were continuous. A statistical analysis was performed using R software (nortest: v1.0-4, dplyr: v1.2.4, ggplot2: v3.5.1) to compare baseline characteristics and to assess spearman correlations among the collected demographic features and hematological parameters.

### 2.3 Feature screening

#### 2.3.1 Least absolute shrinkage and selection operator (LASSO) regression

The LASSO regression method imposes a penalty function to shrink the coefficients of variables toward zero. This process enhances model generalizability by reducing overfitting and alleviating the effects of multicollinearity (27). As a result, LASSO regression improves predictive performance and model interpretability through the selection of the most pertinent risk factors, leading to an efficient and robust predictive model (28). We utilize the glmnet package in R for LASSO regression. A total of 21 hematological parameters from all patients were included in the LASSO regression analysis. The LASSO regression analysis employed a binomial model due to the binary nature of the grouping variable. All predictor variables were standardized before being entered into a binomial LASSO model. The tuning parameter (*λ*) was selected through 10-fold cross-validation implemented via the cv.glmnet function. In brief, the dataset was randomly divided into ten equal parts. For each λ value generated across a logarithmic sequence, the model was trained on nine folds and evaluated on the remaining fold. This process was repeated across all folds, and the average cross-validated deviance was calculated for every candidate *λ*. The optimal penalty parameter (*λ*) was determined using 10-fold cross-validation. For each candidate λ value, the cross-validated mean squared error (MSE) was calculated, and the *λ* that minimized the MSE (λ_min) was selected. We also recorded the λ falling within one standard error of the minimum (λ_1se) and displayed both values in the cross-validation plot. Using the selected optimal λ, the LASSO model was finally refitted:

glmnet (x_train, y_train, family = “binomial,” alpha = 1, lambda = best_lambda)

Predictor variables with non-zero coefficients in the penalized model are retained as the variables selected by LASSO.

#### 2.3.2 Logistic regression

As a form of generalized linear regression, logistic regression finds widespread application in diverse fields such as data mining, automated disease diagnosis, and economic forecasting (29). This approach allows for the identification of disease-related risk factors, thereby enabling the estimation of disease probability based on these identified factors (30). Factors screened by LASSO regression were further used in backward stepwise multivariate logistic regression analysis, ultimately identifying risk factors with *p* < 0.05. The main package versions involved were: pROC: 1.18.5, rms: 6.7.1, forestploter: 1.1.2, ResourceSelection: 0.3.6, ggscidca: 0.2.3, rmda: 1.6, dcurves: 0.5.0, dplyr: 1.1.4, MASS: 7.3.60, ggplot2: 3.5.1.

### 2.4 Data division

The enrolled patients were randomly allocated into training and testing sets at a ratio of 7:3 using the scikit-learn package (version 1.1.3).

### 2.5 Classification multi-model integrated analysis

#### 2.5.1 Construction of predictive models

A total of nine ML models were evaluated in this study, including Logistic Regression (LR), eXtreme Gradient Boosting (XGBoost), Light Gradient Boosting Machine (LightGBM), Random Forest (RF), Adaptive Boosting (AdBoost), Gaussian Naïve Bayes (GNB), Multilayer Perceptron (MLP), Support Vector Machine (SVM), and K-Nearest Neighbors (KNN). During the model training process, a 10-fold cross-validation approach was applied to the training dataset, after which the optimal machine learning model was selected based on its evaluated performance on the test set. All models were implemented in R using the following package versions: colorspace: 2.1–0, gridExtra: 2.3, tidyr: 1.3.0, dplyr: 1.1.3, ggplot2: 3.4.4, calibrate: 1.7.7, PRROC: 1.3.1, pROC: 1.18.4, rmda: 1.6.4, class: 7.3–22, nnet: 7.3–19, e1071: 1.7–13, adabag: 4.2, randomForest: 4.7–1.1, lightgbm: 3.3.5 and xgboost:1.7.5.1 for the remaining algorithms.

#### 2.5.2 Model evaluation outcomes

We evaluated model performance using the area under the receiver operating characteristic curve (ROC), accuracy, sensitivity, specificity, positive predictive value (PPV), negative predictive value (NPV), and the F1-score. The primary outcome involved a comparative analysis of ROC curves among the different models to visually determine which model demonstrated superior performance. The ROC curves were computed using R (package pROC, v1.18.4). The clinical applicability and predictive performance of the models were evaluated using decision curve analysis (DCA), calibration curves, and precision-recall (PR) curves. The DCA evaluates the clinical utility of predictive models by quantifying the net benefit of using a model for decision-making against alternative strategies (e.g., treating all or no patients), thus providing a quantitative framework for assessing the model’s practical value in real-world clinical settings (31). The DCA was performed using R software (package rmda, v1.6.4). Calibration curves were plotted to evaluate the reliability of the model’s predictions, that is, to assess the agreement between the predicted probabilities and the actual observed event frequencies (32). The calibration curves for all nine models were constructed using R software (calibrate v1.7.7). The PR curve is defined as a critical tool for evaluating the performance of binary classifiers, especially on imbalanced datasets (33). The PR curves for all nine models were generated using R software (PRROC v1.3.1).

#### 2.5.3 Evaluation of the optimal model

A 10-fold cross-validation was performed on the training set. Subsequently, ROC curves were generated for the training set and test set based on the 10 cross-validated iterations. The learning curve is a graphical tool used to evaluate the training dynamics and generalization capability of a model. It illustrates the trajectory of model performance as a function of the training dataset size or the number of iterations, thereby aiding in the diagnosis of issues such as underfitting, overfitting, or insufficient training data (34). All the aforementioned steps were visualized using R (e1071: v1.7, caret: v6.0).

#### 2.5.4 SHAP interpretability analysis

The SHAP serves to interpret predictions from ML models. It operates by assigning each feature an importance value for a specific prediction, attributing the model’s output to the additive contribution of its input features, which improves the transparency of the decision process (35). Additionally, SHAP summary plots for individual instances facilitate the interpretation of feature-specific contributions to predictions for each sample. This approach allows for the identification of features exerting positive or negative influences on the prediction of pneumoconiosis, while the distribution of SHAP values highlights which features have the most significant impact on the model’s output. SHAP analysis was implemented using R (fastshap v0.1.0) to generate visualizations of feature-specific SHAP values, interpretive plots illustrating feature importance and contributions, and explanations of model predictions through the analysis of individual instance-level contributions.

### 2.6 Statistical analysis

All variables in this study were continuous. Normally distributed variables are presented as mean ± standard deviation (SD), while non-normally distributed continuous variables are reported as median [interquartile range (IQR); Q1–Q3]. Subsequently, an independent samples t-test was employed to compare normally distributed data between the two groups. In contrast, the Mann–Whitney U test was used for comparisons involving non-normally distributed data. A *p*-value of less than 0.05 was considered statistically significant. All data analyses in this study were performed using R software (version 4.5.1).

## 3 Results

### 3.1 Comparison of baseline data

**Figure 1:**
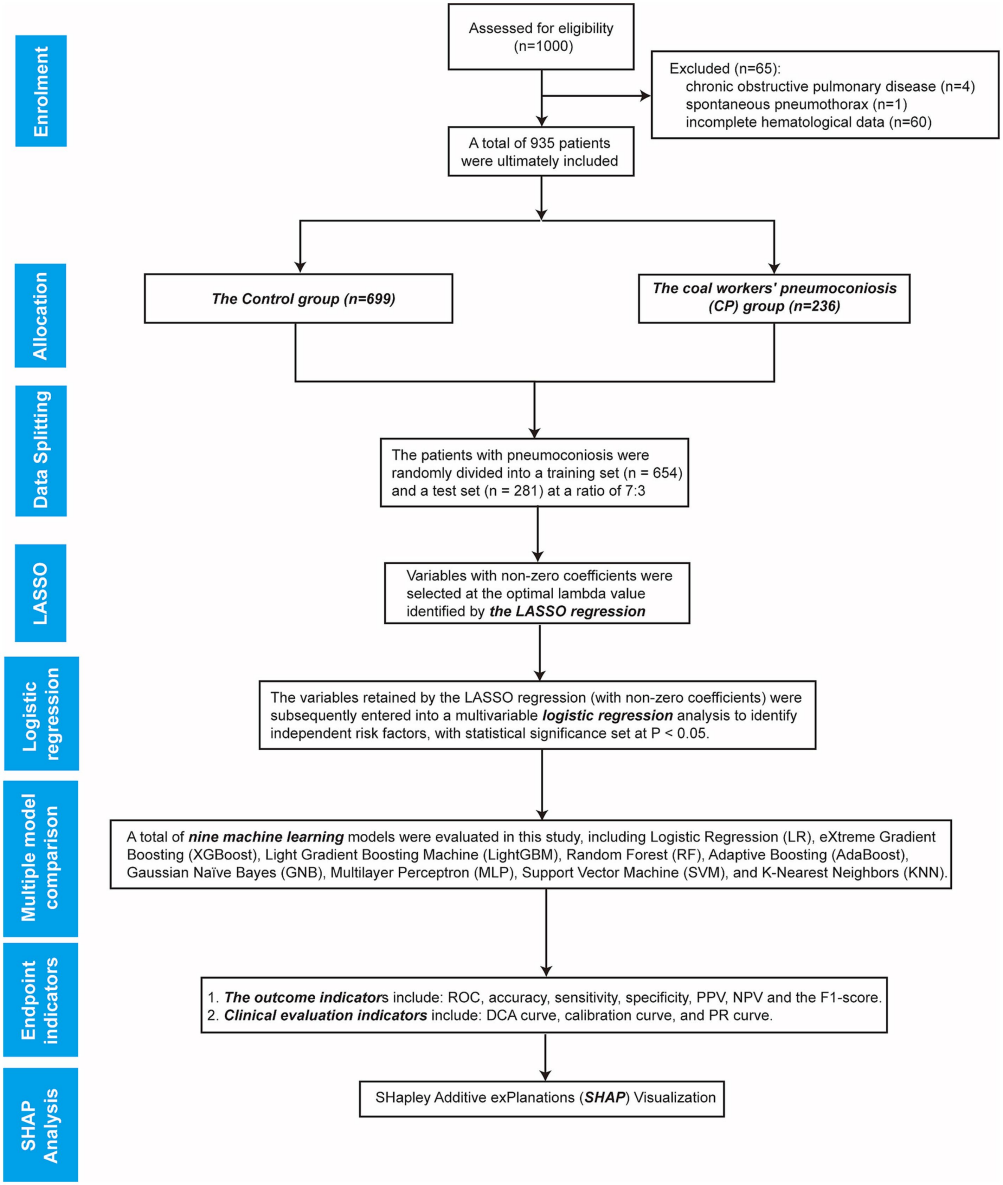
Research flowchart.

**Figure 2:**
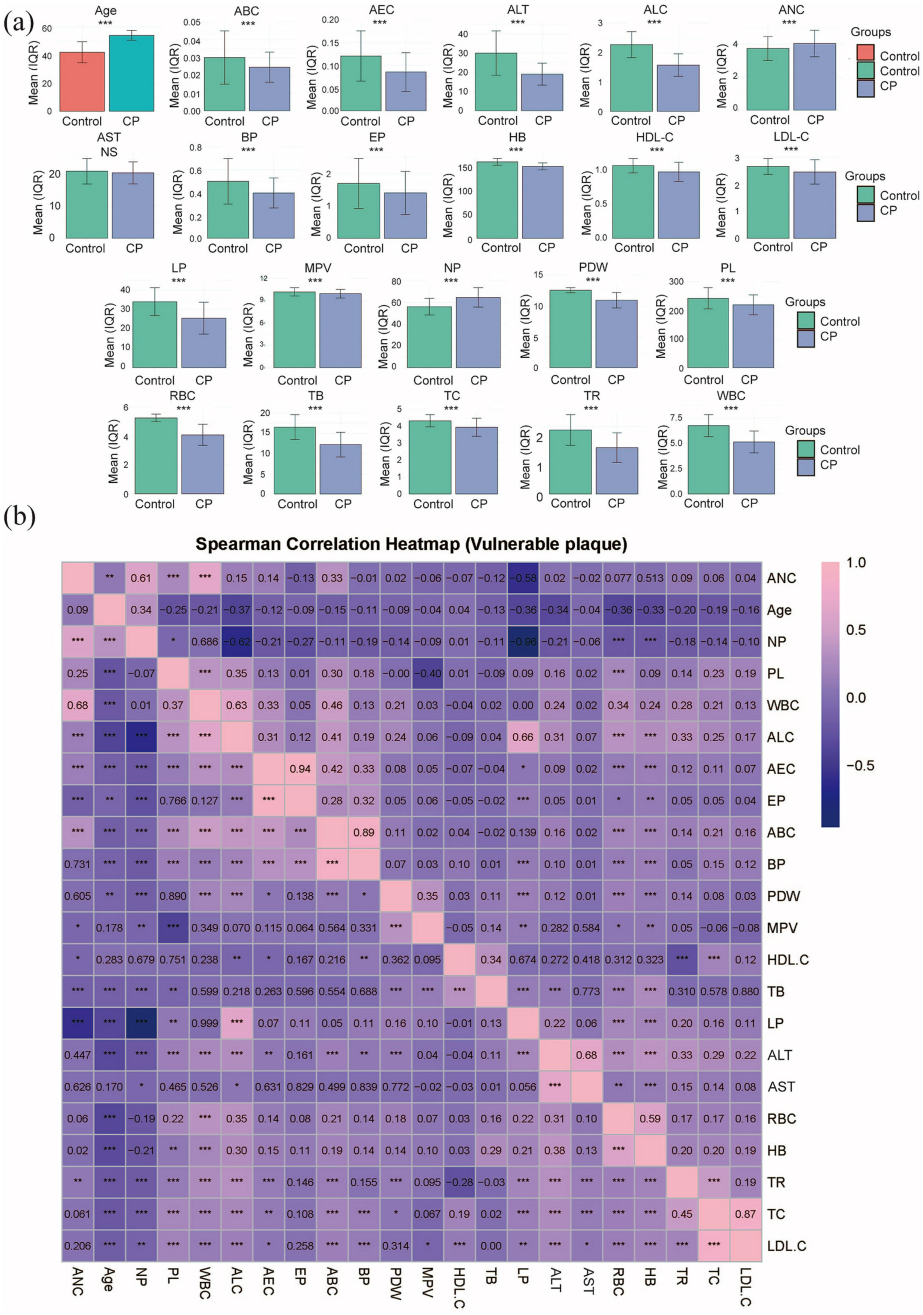
Profiles of 22 demographic and hematological characteristics. **(a)** Bar graph showing the differences for all indices, with significance denoted as *** for *p* < 0.001 and ‘NS’ for non-significant. **(b)** Inter-variable associations are depicted by a Spearman correlation heatmap.

In this study, a total of 1,000 coal miners who underwent occupational health examinations at our hospital’s Department of Occupational Disease Prevention and Control were initially enrolled. Of these, 65 patients with pneumoconiosis were excluded: 5 due to severe complications (4 with chronic obstructive pulmonary disease (COPD) and 1 with spontaneous pneumothorax), and the remaining 60 due to incomplete hematological data. A total of 935 patients were ultimately included in the study. The specific flowchart of this study is shown in Figure 1. Among them, 699 were assigned to the Control group and 236 to the CP group. Comparative analysis of baseline characteristics between the two groups revealed statistically significant differences (*p* < 0.05) in the following parameters: Age, RBC, PL, HB, ANC, NP, ALC, LP, AEC, EP, ABC, BP, PDW, ALT, WBC, MPV, TC, TR, HDL-C, LDL-C, and TB. In contrast, no significant difference was observed in AST (*p* = 0.191), as summarized in Table 1 and Figure 2a. To assess the potential confounding effect of Age, which differed significantly between groups, we generated a Spearman correlation heatmap across all variables. The analysis demonstrated consistently low correlations between age and the 21 hematological indices (the strongest association was Age-ALC, *ρ* = −0.37, *p* < 0.001), as depicted in Figure 2b. Therefore, we concluded that the observed group differences in hematological parameters are not substantially confounded by Age.

**Table 1:** Baseline characterization and comparison.

Variable	ALL (*N* = 935)	Control (*N* = 699)	CP (*N* = 236)	*p*
Age	47.00 (39.00–55.00)	42.00 (34.50–49.50)	54.00 (50.50–57.50)	< 0.001
WBC	6.360 [5.284; 7.680]	6.74 (5.72–7.88)	5.13 (4.04–6.18)	< 0.001
RBC	5.140 [4.731; 5.470]	5.24 (4.98–5.51)	4.07 (3.5–4.95)	< 0.001
PL	236.000 [202.000; 277.667]	242 (209–283)	219.42 (187.83–258.12)	< 0.001
HB	159.000 [152.000; 166.250]	161 (155–169)	151.42 (144.25–159)	< 0.001
ANC	3.810 [3.140; 4.650]	3.73 (3.1–4.56)	4.03 (3.35–4.96)	< 0.001
NP	58.100 [52.000; 64.200]	56.21 ± 7.73	64.69 ± 8.87	< 0.001
ALC	2.110 [1.630;2.620]	2.27 (1.88–2.76)	1.57 (1.24–2)	< 0.001
LP	31.872 (8.341)	34.01 ± 7.22	25.54 ± 8.22	< 0.001
AEC	0.110 [0.070; 0.170]	0.12 (0.07–0.18)	0.09 (0.06–0.14)	< 0.001
EP	1.600 [1.000; 2.517]	1.7 (1.1–2.65)	1.41 (0.87–2.2)	< 0.001
ABC	0.030 [0.020; 0.040]	0.03 (0.02–0.05)	0.02 (0.02–0.04)	< 0.001
BP	0.461 [0.300; 0.600]	0.5 (0.3–0.7)	0.4 (0.29–0.55)	< 0.001
PDW	12.500 [11.900; 13.000]	12.6 (12.2–13)	10.97 (9.97–12.47)	< 0.001
ALT	27.000 [19.000; 40.000]	30 (22–45)	19.02 (14.57–26)	< 0.001
AST	21.000 [17.925; 26.000]	21 (18–26)	20.5 (17.67–24.53)	0.1908
MPV	10.167 [9.600; 10.800]	10.2 (9.7–10.8)	9.96 (9.45–10.63)	< 0.001
TC	4.250 [3.848; 4.670]	4.31 (3.99–4.71)	3.93 (3.38–4.45)	< 0.001
TR	1.810 [1.335; 2.335]	1.91 (1.52–2.43)	1.38 (1.05–1.93)	< 0.001
HDL.C	1.047 [0.930; 1.167]	1.06 (0.96–1.17)	0.97 (0.86–1.14)	< 0.001
LDL.C	2.639 [2.310;2.980]	2.66 (2.41–3)	2.46 (2.04–2.94)	< 0.001
TB	15.246 [12.303; 18.839]	16.6 (13.64–19.78)	12.26 (9.45–15.57)	< 0.001

### 3.2 Screening of characteristic factors for risk of CP

**Figure 3:**
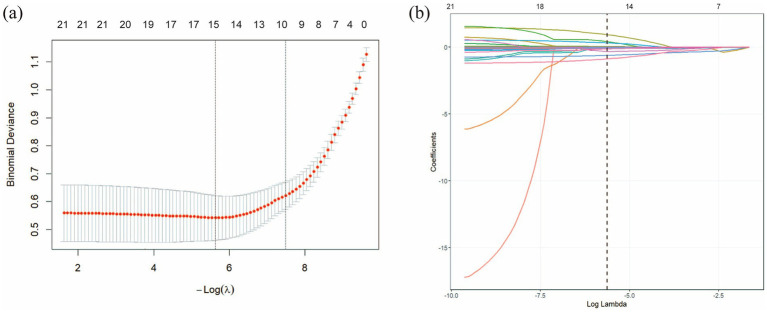
LASSO regression analysis was used to select characteristic factors. **(a)** The trajectory of variable selection in the LASSO regression model is illustrated. The optimal *λ* value resulted in 15 non-zero coefficients. **(b)** In the LASSO model, the coefficient path for each feature was plotted against the sequence of log (λ). The optimal lambda value (λ.min = 0.003575) is indicated by a vertical black dashed line. λ.min: the minimum mean squared error λ.

The patients with pneumoconiosis were randomly divided into a training set (*n* = 654) and a test set (*n* = 281) at a ratio of 7:3. Final statistical analysis indicated no significant differences in baseline characteristics between the training and test sets (*p* > 0.05, Table 2), confirming that the distribution of variables was balanced and would not introduce bias in model development. The results of the LASSO regression are presented in Figure 3a, which illustrates the trajectory of variable coefficients against varying *λ*, with each line representing the coefficient path of an individual variable. As shown in Figure 3b, the model corresponding to the minimum mean squared error (λ.min = 0.003575) retained 15 variables: WBC, RBC, PL, HB, ANC, LP, BP, PDW, ALT, AST, MPV, TC, TR, LDL-C, and TB. In contrast, the one-standard-error rule (λ.1se = 0.1587) selected a more parsimonious set of 9 variables. To retain a more comprehensive set of features for subsequent analysis, the lambda value of 0.003575 (λ.min) was chosen as the optimal penalty parameter. To enhance model interpretability and mitigate the risk of overfitting associated with complex feature combinations, binary logistic regression was performed to evaluate the impact of these 15 variables on pneumoconiosis patients. As shown in Table 3, 6 indicators were identified as potential risk factors for pneumoconiosis (*p* < 0.05), including WBC, PDW, TB, ANC, ALT and AST.

**Table 2:** Training set and test set variability analysis.

Variable	ALL (*N* = 935)	Training set (*N* = 654)	Testing set (*N* = 281)	*p*
WBC	6.36 (5.28–7.68)	6.4 (5.29–7.75)	6.24 (5.27–7.54)	0.4312
RBC	5.14 (4.73–5.47)	5.14 (4.73–5.47)	5.15 (4.76–5.46)	0.9719
PL	236 (202–277.67)	235 (204.88–277)	237.11 (198–278.5)	0.815
HB	159 (152–166.25)	159 (152–166)	159 (152–167)	0.6392
ANC	3.81 (3.14–4.65)	3.84 (3.15–4.72)	3.74 (3.12–4.56)	0.5709
NP	58.1 (52–64.2)	58.1 (51.88–63.91)	58.1 (52.25–64.4)	0.8044
ALC	2.11 (1.63–2.62)	2.11 (1.62–2.64)	2.11 (1.68–2.6)	0.9105
LP	31.87 ± 8.34	31.84 ± 8.49	31.95 ± 8	0.8405
AEC	0.11 (0.07–0.17)	0.11 (0.07–0.17)	0.1 (0.06–0.16)	0.5628
EP	1.6 (1–2.52)	1.64 (1–2.6)	1.6 (1–2.5)	0.5151
ABC	0.03 (0.02–0.04)	0.03 (0.02–0.04)	0.03 (0.02–0.04)	0.4019
BP	0.46 (0.3–0.6)	0.5 (0.3–0.61)	0.4 (0.3–0.6)	0.7595
PDW	12.5 (11.9–13)	12.5 (11.9–12.96)	12.5 (11.9–13.07)	0.8194
ALT	27 (19–40)	27 (19–42)	25.61 (19–37)	0.2735
AST	21 (17.92–26)	21 (18–26)	20.74 (17–25)	0.3012
MPV	10.17 (9.6–10.8)	10.2 (9.6–10.8)	10.1 (9.6–10.7)	0.3079
TC	4.25 (3.85–4.67)	4.25 (3.87–4.65)	4.25 (3.76–4.74)	0.891
TR	1.81 (1.33–2.33)	1.82 (1.36–2.35)	1.76 (1.23–2.26)	0.2638
HDL.C	1.05 (0.93–1.17)	1.04 (0.93–1.16)	1.06 (0.95–1.17)	0.4158
LDL.C	2.64 (2.31–2.98)	2.63 (2.32–2.94)	2.65 (2.28–3.04)	0.5257
TB	15.7 (12.4–19.1)	15.65 (12.5–18.88)	15.9 (11.95–19.8)	0.9565

**Table 3:** Logistic regression analysis results.

Variable	Estimate	Std. error	OR	*p*
ALT	−0.07273	0.016942	0.929854 (0.90–0.96)	<0.001
ANC	1.327743	0.287358	3.772518 (2.19–6.77)	<0.001
AST	0.07326	0.018336	1.07601 (1.03–1.12)	<0.01
BP	0.813773	0.569393	2.256406 (0.74–6.91)	>0.05
HB	−0.02455	0.014146	0.975747 (0.95–1.00)	>0.05
LDL-C	−0.4315	0.809333	0.649531 (0.14–3.27)	>0.05
LP	−0.03984	0.030177	0.96094 (0.91–1.02)	>0.05
MPV	0.458765	0.213295	1.582118 (1.04–2.42)	>0.05
PDW	−0.72799	0.136116	0.482879 (0.37–0.62)	<0.001
PL	−0.00255	0.003703	0.997448 (0.99–1.00)	>0.05
RBC	0.001055	0.002182	1.001055 (1.00-NA)	>0.05
TB	−0.12984	0.027696	0.878236 (0.83–0.92)	<0.001
TC	−0.03103	0.732642	0.969442 (0.22–3.95)	>0.05
TR	−0.24987	0.21574	0.778904 (0.50–1.18)	>0.05
WBC	−1.13175	0.180235	0.322468 (0.22–0.45)	<0.001

### 3.3 Comprehensive analysis results of classification and multi-model

**Figure 4:**
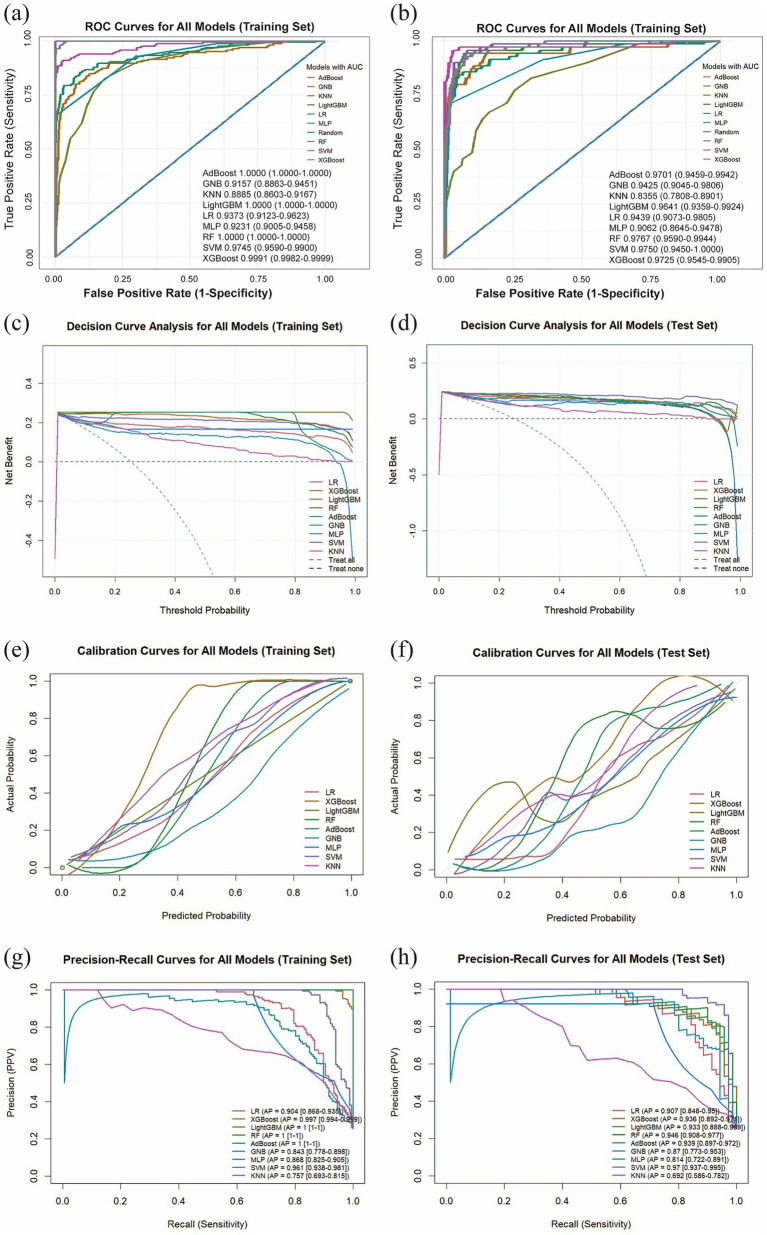
Comprehensive analysis of machine learning model performance. **(a)** ROC curves and AUC values for the training set. **(b)** ROC curves and AUC values for the test set. **(c)** DCA for the training set. The diagonal dashed line assumes that all patients have pneumoconiosis, while the horizontal dashed line represents the strategy of treating no patients. The solid lines correspond to the nine predictive models. **(d)** DCA for the training set. **(e)** Calibration curves for the training set. The *x*-axis represents the mean predicted probability, and the y-axis denotes the actual observed probability. The diagonal line serves as the reference (ideal calibration). The smooth solid lines are fitted curves for each model; closer proximity to the reference line and smaller values in parentheses indicate better calibration accuracy. **(f)** Calibration curves for the test set. **(g)** PR curve and average precision (AP) for the training set. The *y*-axis indicates precision, and the *x*-axis represents recall. If the PR curve of one model is entirely enclosed by that of another, the latter demonstrates superior performance. Higher AP values reflect better model performance. Distinct colors in the figure represent each corresponding model. **(h)** PR curve and AP for the test set.

We utilized the final 6 risk variables to develop nine different machine learning models. The evaluation metrics for each model are shown in Figure 4a and Table 4. The AUC (95%CI) values for the nine models in the training set are 0.9373 (0.9123–0.9623) for LR, 0.9991 (0.9982–0.9999) for XGBoost, 1.0000 (1.0000–1.0000) for LightGBM, 1.0000 (1.0000–1.0000) for RF, 1.0000 (1.0000–1.0000) for AdBoost, 0.9157 (0.8863–0.9451) for GNB, 0.9231 (0.9005–0.9458) for MLP, 0.9745 (0.9590–0.9900) for SVM, and 0.8885 (0.8603–0.9167) for KNN. The AUC values of 1.000 achieved by LightGBM, RF and AdBoost indicate perfect predictive accuracy on the training dataset. As shown in Figure 4b and Table 4, the AUC (95%CI) values of the nine models in the test set are 0.9439 (0.9073–0.9805) for LR, 0.9725 (0.9545–0.9905) for XGBoost, 0.9641 (0.9359–0.9924) for LightGBM, 0.9767 (0.9590–0.9944) for RF, 0.9701 (0.9459–0.9942) for AdBoost, 0.9425 (0.9045–0.9806) for GNB, 0.9062 (0.8645–0.9478) for MLP, 0.9750 (0.9450–1.0000) for SVM, and 0.8355 (0.7808–0.8901) for KNN. Among all models, LightGBM (AUC = 0.9767) and SVM (AUC = 0.9750) achieved the highest predictive performance on both the test sets, outperforming the conventional logistic regression model. The SVM model demonstrated a minimal performance gap in the AUC between the training and test sets, with superior predictive accuracy observed in the test set. This indicates that the model generalizes effectively and exhibits remarkable stability. We also compared model performance based on the following criteria: accuracy, sensitivity, specificity, PPV, NPV, and the F1-score. As summarized in Table 4, SVM performs the best on these evaluation metrics. Based on our comparative analysis, the SVM model demonstrated superior predictive performance for identifying patients with pneumoconiosis. Further clinical utility assessment was conducted by analyzing DCA, calibration curves, and PR curves across all nine models. As shown in Figures 4c,d, the DCA of test set indicated that both SVM exhibited better clinical utility compared to the other models. This suggests the SVM model enhanced applicability for clinical decision-making. The calibration curves presented in Figures 4e,f demonstrated that SVM provided reliable predictive performance. According to the PR curves in Figures 4g,h, SVM achieved the highest average precision (AP = 0.97, 0.937–0.995) in the test set. Based on these comprehensive findings, SVM was determined to be the optimal model.

**Table 4:** Comparison of training and test set results for 9 models.

Model	Dataset Splitting	AUC	Accuracy	Sensitivity	Specificity	PPV	NPV	F1
LR	Training	0.9373 (0.9123–0.9623)	0.9207	0.7711	0.9714	0.9014	0.9261	0.8312
	Test	0.9439 (0.9073–0.9805)	0.9211	0.8143	0.9569	0.8636	0.9390	0.8382
XGBoost	Training	0.9991 (0.9982–0.9999)	0.9787	0.9157	1.0000	1.0000	0.9722	0.9560
	Test	0.9725 (0.9545–0.9905)	0.9319	0.8143	0.9713	0.9048	0.9398	0.8571
LightGBM	Training	1.0000 (1.0000)	1.0000	1.0000	1.0000	1.0000	1.0000	1.0000
	Test	0.9641 (0.9359–0.9924)	0.9283	0.8000	0.9713	0.9032	0.9355	0.8485
RF	Training	1.0000 (1.0000)	1.0000	1.0000	1.0000	1.0000	1.0000	1.0000
	Test	0.9767 (0.959–0.9944)	0.9283	0.8143	0.9665	0.8906	0.9395	0.8507
AdBoost	Training	1.0000 (1.0000)	1.0000	1.0000	1.0000	1.0000	1.0000	1.0000
	Test	0.9701 (0.9459–0.9942)	0.9355	0.8286	0.9713	0.9062	0.9442	0.8657
GNB	Training	0.9157 (0.8863–0.9451)	0.8796	0.8133	0.9020	0.7377	0.9345	0.7736
	Test	0.9425 (0.9045–0.9806)	0.8889	0.8714	0.8947	0.7349	0.9541	0.7974
MLP	Training	0.9231 (0.9005–0.9458)	0.9131	0.6566	1.0000	1.0000	0.8958	0.7927
	Test	0.9062 (0.8645–0.9478)	0.9068	0.6857	0.9809	0.9231	0.9031	0.7869
SVM	Training	0.9745 (0.959–0.99)	0.9527	0.8133	1.0000	1.0000	0.9405	0.8970
	Test	0.9750 (0.945–1)	0.9570	0.8714	0.9856	0.9531	0.9581	0.9104
KNN	Training	0.8885 (0.8603–0.9167)	0.8323	0.4458	0.9633	0.8043	0.8369	0.5736
	Test	0.8355 (0.7808–0.8901)	0.8100	0.4286	0.9378	0.6977	0.8305	0.5310

### 3.4 Evaluation of the optimal model (SVM)

**Figure 5:**
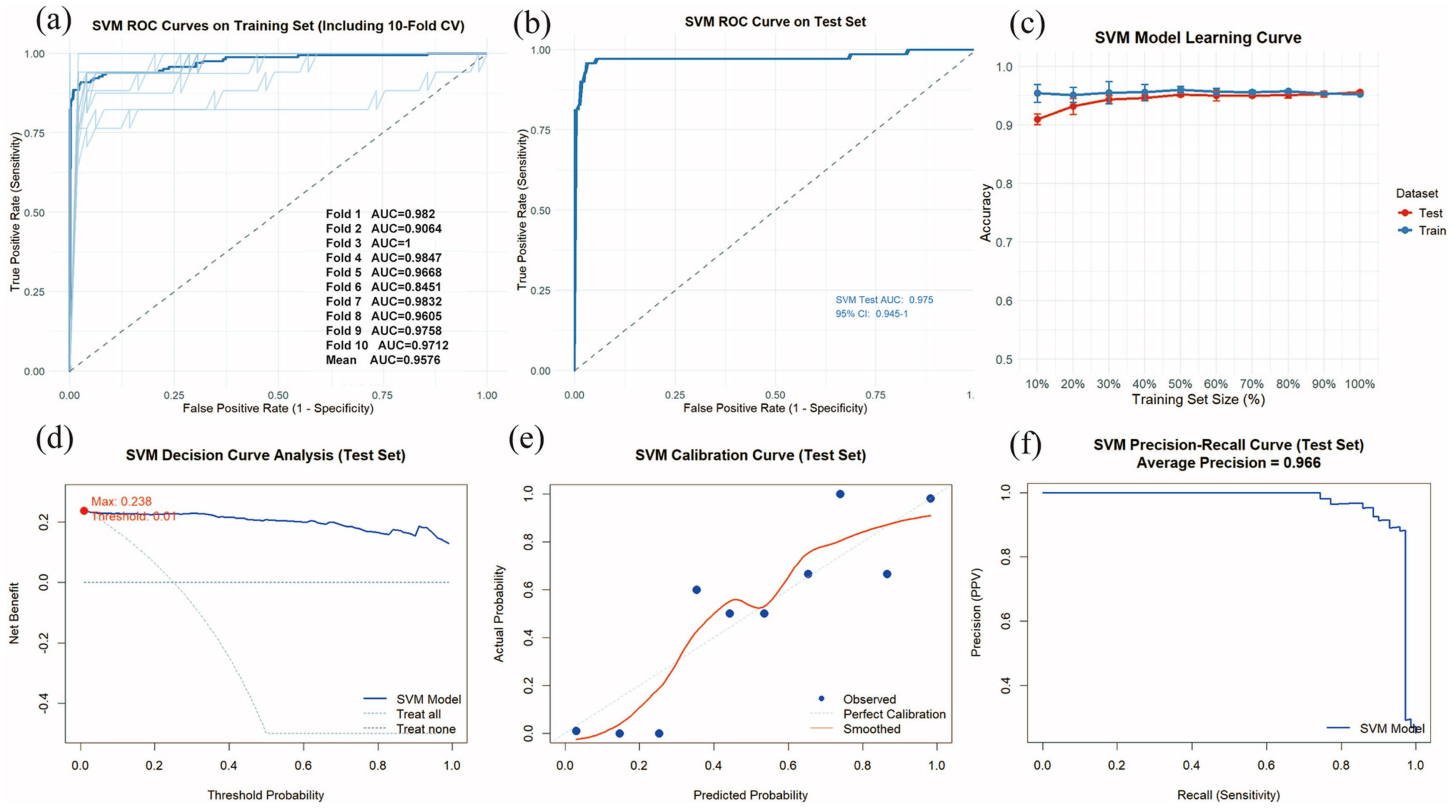
Evaluation of the SVM model. **(a)** Training set ROC curves and AUC based on 10-fold cross-validation. The dark blue line represents the mean AUC value, while the light blue lines depict the results of the 10 individual cross-validation folds. **(b)** ROC curve and AUC for the test set. **(c)** The learning curves are shown for the training set (blue solid line) and the validation set (red solid line). **(d)** Decision curve analysis (DCA) for the test set. **(e)** Calibration curves for the test set. **(f)** PR curve and AP for the test set.

The SVM model was constructed using the 6 statistically significant variables identified by logistic regression: WBC, PDW, TB, ANC, ALT and AST. A 10-fold cross-validation was performed on the training set, with the validation subset comprising 10% of the training data in each fold. The validation results are presented in Figures 5a,b. The model achieved an average AUC of 0.9576 in 10-fold cross-validation on the training set, and 0.9750 (0.9450–1.0000) on the test set. Given that the performance of the test set under the AUC metric does not exceed that of the test set or exceeds it by less than 10%, it can be considered that the model has fit successfully. Furthermore, the learning curves of the training and test sets converged to a comparable performance level, indicating a reliable model without signs of overfitting, as illustrated in Figure 5c. Therefore, the SVM model can be considered suitable for classification tasks on this dataset. The decision curve for the test set (Figure 5d) indicated an optimal decision threshold of 0.01 for the SVM model, corresponding to a clinical scenario wherein intervention is warranted when a patient’s predicted probability of pneumoconiosis exceeds 1%. This indicates that SVM has a wide range of clinical applications. The maximum net benefit achieved by the model was 0.238, demonstrating that, at this threshold, using the SVM model to guide clinical decisions yields a net gain of approximately 24 true positive cases per 100 patients compared to the “treat-none” strategy. These results confirm the potential clinical utility of the SVM model for predicting pneumoconiosis. Figures 5e,f present the calibration curve and PR curve for the SVM model, respectively. The calibration curve, after smoothing, closely aligns with the diagonal line of perfect calibration, indicating a strong agreement between the predicted probabilities of pneumoconiosis and the observed event frequencies. Furthermore, the PR curve yielded an average precision (AP) score of 0.966 on the test set, further affirming the model’s exceptional capability in accurately identifying true pneumoconiosis patients.

### 3.5 SHAP-based model interpretability analysis

**Figure 6:**
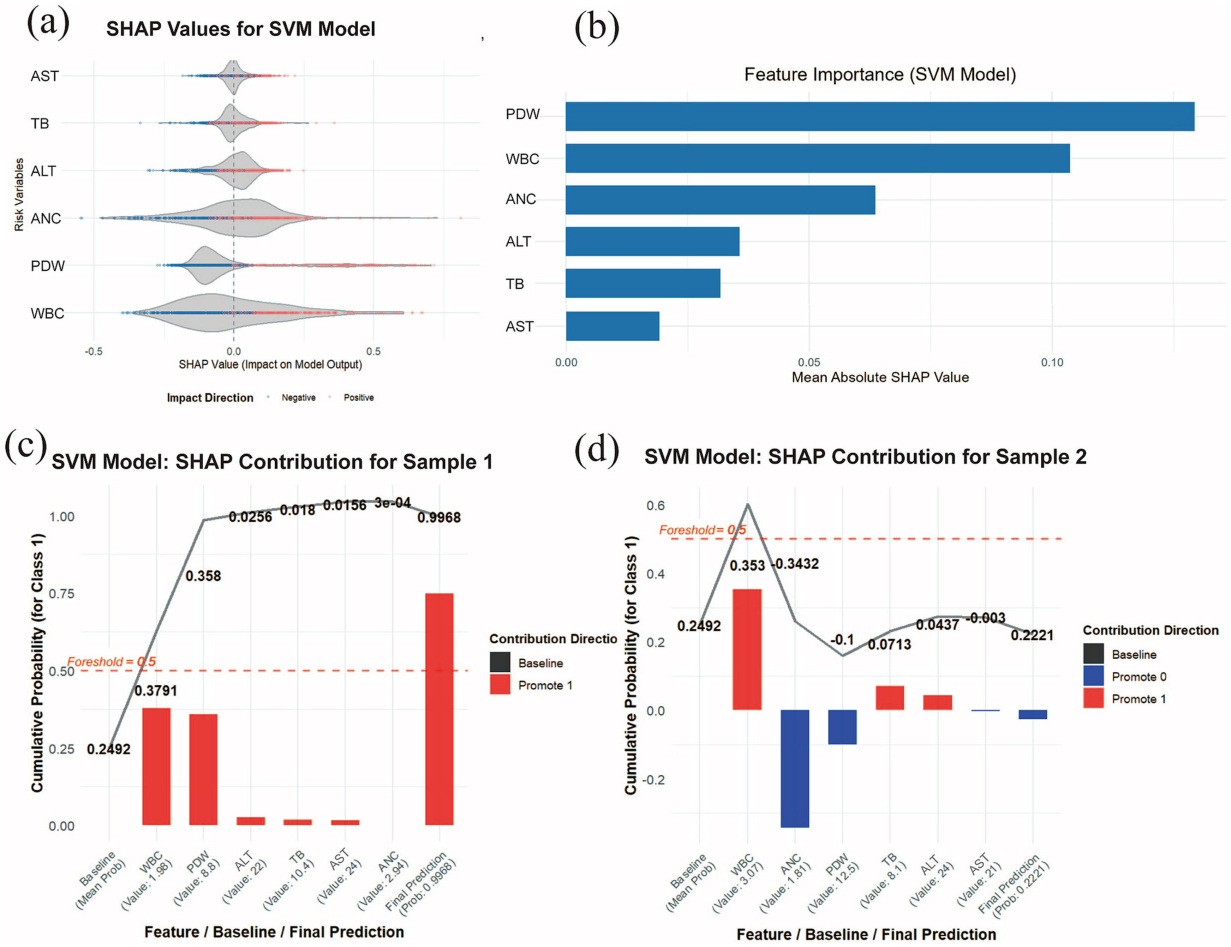
SHAP interpretability analysis. **(a)** Violin plot of SHAP values for the SVM model. The central bulge of each violin illustrates the density distribution of a feature’ s contribution direction. A SHAP value greater than zero indicates a positive contribution to the disease prediction, whereas a value less than zero signifies a negative contribution. **(b)** Feature importance ranking derived from SHAP analysis. The bar chart depicts the relative importance of each covariate in the final predictive model. **(c)** Individual contribution plot for a patient without pneumoconiosis and **(d)** individual contribution plot for a patient with pneumoconiosis. Red indicates positive SHAP values (increasing prediction risk), while blue represents negative SHAP values (decreasing prediction risk).

By examining the distribution of SHAP values for each risk indicator, it is possible to identify which features exert a significant influence on the prediction outcomes. This study evaluated the 6 most important features associated with the development of pneumoconiosis in patients. As shown in Figure 6a, the SHAP violin plots for the six risk indicators revealed that TB, PDW, and WBC exerted negative effects against the development of pneumoconiosis, whereas ALT and ANC were identified as (positive factors. This suggests that lower levels of TB, PDW, and WBC, coupled with higher levels of ALT and ANC, are associated with an increased risk of disease progression. In contrast, AST, with its SHAP values distributed near zero, had a comparatively minimal impact. As displayed in Figure 6b, the *Y*-axis lists the seven most important features associated with pneumoconiosis development in patients, ranked in descending order of importance (from top to bottom). The *X*-axis represents the mean absolute SHAP value (mean |SHAP value|), which reflects the average magnitude of each feature’s contribution to the model output. A larger value indicates a more substantial impact on the model’s predictions. The length of the blue bars illustrates the relative importance ranking of these 6 features in relation to pneumoconiosis formation. The results indicated that PDW, WBC, and ANC represented the three most influential factors contributing to pneumoconiosis development. To enhance the interpretability of the model’s decision-making process at the individual level, a detailed explainable analysis was conducted on two representative samples (Figures 6c,d). This analysis visualizes the contribution magnitude and direction (with red bars representing positive contributions and blue bars representing negative contributions) of each feature toward the prediction of pneumoconiosis for each selected patient, along with the final predicted value, thereby facilitating an assessment of individual risk for pneumoconiosis development. Two representative samples were analyzed: one from a patient without pneumoconiosis and the other from a confirmed case. The SVM model assigned a high predicted probability of 0.9968 to the latter sample. The prediction was primarily driven by WBC and PDW, which were the strongest positive contributors with SHAP values of 0.3791 and 0.358, respectively. For Sample 2, the SVM model assigned a low prediction probability of 0.2221, indicating a low likelihood of pneumoconiosis. This outcome was primarily driven by the opposing effects of key features: while WBC exerted the strongest positive influence (SHAP value = 0.353), its effect was counteracted by negative contributions from ANC (SHAP value = −0.3432) and PDW (SHAP value = −0.1). The primary features contributing to this prediction include low WBC, low PDW and high ANC. However, the two indicators that had the least impact on individual samples were ALT and AST.

## 4 Discussion

In this study, 6 risk factors related to the occurrence of pneumoconiosis were screened out by collecting blood indicators from all coal miner patients using lasso regression and logistic regression. Nine ML algorithm models were evaluated to develop a clinical prediction model for coal workers’ pneumoconiosis. By comparing the performance and clinical applicability of all models, the results show that SVM shows the best performance. In the best model validation, an AUC of 0.9745 was achieved in the training set and 0.9750 was achieved in the validation set, and excellent clinical predictive power was shown in the DCA plot. To improve the interpretability of our ML models, we report SHAP values and a range of highly influential features.

The diagnosis of pneumoconiosis relies on a combination of clinical assessments. It primarily depends on alterations in lung function, assessed by pulmonary function tests (PFTs), and structural changes in the lungs, identified through radiological imaging such as chest computed tomography (CT), radiography, or histopathological findings from lung biopsy. This diagnostic process is complemented by a confirmed history of occupational exposure to mineral dust, evaluation of clinical manifestations, and an assessment of the individual’s work environment (6, 10, 36, 37). Pneumoconiosis is characterized by a prolonged latency period, during which most patients remain asymptomatic. Clinical manifestations, including dyspnea, cough, sputum production, and chest pain, typically emerge only after sustained exposure to mineral dust. By the time of diagnosis, the disease is often irreversible and complicated by comorbidities such as tuberculosis, emphysema, or COPD (38). The characteristically delayed onset of clinical manifestations in pneumoconiosis, combined with the complexity of its diagnostic workup, poses a significant challenge to early and accurate detection. Consequently, the disease is frequently diagnosed at an advanced, irreversible stage for which no effective curative treatments are currently available (6). The overlapping clinical and radiological presentations among pneumoconiosis, lung cancer, tuberculosis, and sarcoidosis often preclude a definitive diagnosis based on conventional imaging alone. This diagnostic ambiguity underscores the critical importance of early detection and timely intervention for effective patient management (39). While techniques like high-resolution computed tomography (HRCT), chest radiography (CR), electrical impedance tomography (EIT), and magnetic pulmonography can facilitate the early diagnosis of chronic lung diseases, their accessibility remains limited. This is particularly evident in smaller hospitals due to a lack of necessary infrastructure and the prohibitively high costs associated with these technologies (40–43). The development and application of novel diagnostic biomarkers and therapeutic targets are therefore imperative. Recent advances in elucidating the pathogenesis of pneumoconiosis, characterized by lung inflammation and progressive fibrosis, have identified promising biomarkers. Specific cytokines, inflammatory factors such as interleukin-8 (IL-8) and prostaglandin D (PGD), transcription factors, and proteins have emerged as potential candidates for new diagnostic tools and treatments (44–47). Emerging evidence suggests that genetic biomarkers, particularly microRNAs (miRNAs) and circular RNAs (circRNAs), hold considerable promise for the diagnosis of pneumoconiosis (48–50). However, the clinical utility of these methods is often limited by their high cost. To circumvent these limitations, this study leveraged routinely collected blood indicators from physical examinations of coal miners to identify cost-effective biomarkers associated with the development of pneumoconiosis.

Lung inflammation is an early typical pathological change of pneumoconiosis (51). Dust precipitation in the lungs activates an immune response by recruiting and activating inflammatory cells and secreting relevant inflammatory factors that activate fibroblasts and promote fibrosis progression. The clearance and defense mechanisms of the respiratory system in pneumoconiosis patients are severely impaired, and co-infection can lead to further increases in inflammation levels in pneumoconiosis patients (6). Studies have found that blood inflammation index may be an indicator to assist in the early diagnosis and assessment of pneumoconiosis (52). This study identified six hematological biomarkers associated with the development of pneumoconiosis: WBC, PDW, TB, ANC, ALT, and AST.

WBCs are a key component of the body’s immune system and contain a variety of cell types, including neutrophils and lymphocytes (53). The findings of this study indicate a significant association between decreased WBC count and the occurrence of pneumoconiosis. As shown in Table 1, the WBC count was significantly lower in the CP group compared to the Control group. SHAP analysis showed that WBC count was the most closely related feature to pneumoconiosis, which may be due to the involvement of WBC in the early inflammatory response of pneumoconiosis. In patients with stage I CWP, peripheral leukocyte subsets exhibit a complex pattern of alterations. Eosinophils and basophils tend to be reduced, monocytes are markedly elevated, lymphocytes show a declining trend, and neutrophils are generally increased. As a result, the overall leukocyte count may appear either elevated or reduced, depending on the dominant cell subgroup (54). Consistent with these findings, Guo-kang Sun et al. reported that individuals with silicosis had lower total leukocyte counts than healthy controls, largely attributable to a decrease in lymphocyte numbers (25). This observation aligns with our results, showing reduced leukocyte counts during the early stage of pneumoconiosis. These changes are collectively driven by chronic inflammation, oxidative stress, and immune dysregulation induced by inhaled coal dust particles (55, 56). Activation of alveolar macrophages and the release of pro-inflammatory cytokines play a central role in leukocyte recruitment and differentiation (57). Modulating macrophage polarization has been shown to attenuate silica-induced pro-inflammatory and pro-fibrotic responses, thereby alleviating silicosis (58, 112). As the disease progresses into stages II and III, leukocyte dynamics become even more heterogeneous. In stage II CWP, leukocyte alterations are not characterized by a simple increase or decrease, but rather by shifts in the composition and functional states of different leukocyte subsets. Chronic inflammation, the onset of pulmonary fibrosis, and potential secondary infections or other complications may all contribute to elevated leukocyte levels (59, 60). Sustained cytokine release—such as IL-6, IL-12, and IFN-*γ*—can drive increases in peripheral leukocyte counts, particularly in inflammation-related subsets such as neutrophils and monocytes, in response to persistent pulmonary injury (61). Silica exposure activates the inflammasome, which reduces the population and impairs the function of regulatory T cells (62). This thereby disrupts the balance between Tregs and effector T cells, consequently increasing susceptibility to systemic autoimmune diseases (62). Furthermore, prolonged disease progression leads to a general decline in immune function, often manifested as a reduction in lymphocyte counts, which also contributes to leukopenia in the peripheral blood (63). Impaired autophagic activity in alveolar macrophages may hinder the clearance of coal dust particles and apoptotic cells, thereby amplifying pulmonary inflammation and indirectly influencing leukocyte number and function (64). Genetic susceptibility may further contribute to disease progression; for instance, polymorphisms in the MUC5B promoter region—previously implicated in fibrotic lung diseases—are associated with increased CWP risk and may promote the transition toward fibrosis (65). These observations indicate that inflammatory signaling, autophagy dysregulation, and genetic factors collectively shape leukocyte behavior during disease progression (66, 67). Taken together, these findings underscore the central role of leukocytes throughout the development of CWP. Peripheral WBC do not follow a simple linear pattern of increase or decrease across disease stages; rather, they are influenced by multiple interacting factors. Our results show that leukocyte levels decline during the early phase of CWP, a finding that may hold interpretive value. Further studies are warranted to clarify the overall trajectory of leukocyte changes throughout disease progression.

Neutrophils are important markers of the body’s response to inflammatory infection. SHAP analysis in this study revealed that a higher ANC exerts a positive effect on the development of pneumoconiosis. Consistent with this, as presented in Table 1, the ANC was significantly elevated in the CP group compared to the Control group. Several studies have shown that the number of neutrophils in pneumoconiosis patients is higher than in the normal population. In the study by Y. J. Diao et al. (52), it was found that blood inflammatory markers, including ANC, may be auxiliary predictors of stage I pneumoconiosis and its comorbid lung infections. A study by H. Deng et al. (68) showed that the age, neutrophils, and basophils of CP patients with the same genotype were higher than those in the control group, and the differences were statistically significant (all *p* < 0.05). A study by Iiao et al. (69) found that neutrophils in the pneumoconiosis combined with acute exacerbation of chronic obstructive pulmonary disease (AECOPD) group were significantly higher than those in the AECOPD group (*p* < 0.05). These are consistent with our findings. An elevated neutrophil level contributes to the inflammatory response initiated by coal dust entry into the body. During early-stage pneumoconiosis, coal dust particles act as a direct irritant to lung cells, prompting inflammation (3, 111). Macrophages play a pivotal role in this process by recognizing and engulfing coal dust particles, subsequently releasing a variety of inflammatory mediators, including the chemokine IL-8 and the cytokine TNF-*α* (61). Interleukin-8 (IL-8) is a potent neutrophil-chemoattractant that efficiently recruits circulating neutrophils to sites of inflammation (61). Tumor necrosis factor-α (TNF-α), a key pro-inflammatory cytokine, amplifies the inflammatory response and further promotes neutrophil activation and survival (70). In addition, coal mine dust has been shown to induce upregulation of the NLRP3 inflammasome (71). NLRP3 is a critical component of the innate immune system, and its activation triggers the maturation and release of the pro-inflammatory cytokines IL-1β and IL-18 (72). These cytokines further exacerbate pulmonary inflammation, enhance neutrophil recruitment and activation, and consequently contribute to elevated neutrophil level (72). These findings corroborate our observation of increased neutrophil counts in the early stage of CWP. With continued exposure to coal dust, pulmonary inflammation transitions from an acute to a chronic state, eventually accompanied by tissue remodeling and fibrosis (9). During stage II CWP, systemic inflammation persists, and peripheral neutrophil counts may remain elevated (73). Programmed cell death pathways such as pyroptosis may also act in the peripheral blood of CWP patients; this mode of cell death is typically associated with intense inflammatory responses and may drive neutrophil activation and cytokine release, offering potential therapeutic and diagnostic targets (73). In stage III CWP, fibrotic lesions become more extensive and lung function is considerably impaired (74). Widespread fibrosis and severe structural disruption may permit continued neutrophil involvement in tissue injury and remodeling, thereby sustaining an upward trend in neutrophil abundance (75). Experimental studies further show that the S100a8^hi neutrophil subset is expanded following coal dust exposure, and that the S100a8-specific inhibitor paquinimod ameliorates inflammation and fibrosis while improving lung function by blocking the S100a8–TLR4–macrophage signaling axis (76). Collectively, these findings indicate that neutrophil levels tend to increase as CWP progresses. These results highlight the value of ANC as a promising diagnostic biomarker for monitoring disease progression in pneumoconiosis.

CP is caused by chronic inhalation and deposition of coal particles in the lungs. Studies have reported that reactive oxygen species (ROS) play an important role in the pathogenesis of pneumoconiosis disease. During phagocytosis of inhaled dust particles, ROS are released from alveolar macrophages and leukocytes. During the early stages of pneumoconiosis, inhalation of harmful occupational agents leads to excessive production of reactive oxygen species (ROS) during phagocytosis. The interplay between the immune response and this resultant oxidative stress is implicated in the development of the disease (77). In addition, dust particles themselves produce ROS (78). ROS has been reported to induce platelet activation, which plays a crucial role in the inflammatory process (79, 110). According to previous data, ROS acts as stimulators of platelet activation, adhesion, and aggregation through multiple signaling mechanisms, namely by regulating NO and arachidonic acid metabolism (79). Platelets not only play a central role in hemostasis but are also recognized as key participants in innate immune responses. They contribute to inflammatory processes by transporting mediators, promoting leukocyte adhesion and activation, and forming platelet–leukocyte aggregates (80). PDW is frequently used as an indirect indicator of inflammation, oxidative stress, and platelet activation (81). As CWP progresses to stages II and III, chronic inflammation intensifies, which may further promote platelet activation and result in higher PDW values. This pattern is consistent with the findings of Firat Uygur and Han-Yu-Jie Kang, who reported elevated PDW in patients with advanced pneumoconiosis (77, 82). Moreover, persistent inflammatory activity stimulates the release of multiple cytokines, including TGF-β1, TNF-*α*, MCP-1, and IL-1, whose circulating levels have been associated with the progression of CWP (83, 84). The release of these cytokines may promote the proliferation and differentiation of megakaryocytes in the bone marrow, thereby enhancing platelet production (52). As shown in Table 1, the CP group exhibited a significantly lower PDW compared with the control group. We also observed that the PL in the CP group was markedly reduced relative to the control group (*p* < 0.001). Our findings are inconsistent with the conclusions of the aforementioned studies. Decreases in platelet parameters can be attributed to multiple factors, including immune-mediated thrombocytopenia, heparin-induced thrombocytopenia, and drug-related effects (85, 86). However, given the large sample size, it is unlikely that heparin or medications alone account for the observed reductions. Therefore, the thrombocytopenia observed in pneumoconiosis patients may be associated with immune-related mechanisms. In patients with pneumoconiosis, the ingestion of silica particles by macrophages triggers a robust and sustained inflammatory response and leads to the disruption of immune tolerance (87). This chronic immune activation may lead to the production of antiplatelet antibodies (anti-GPIIb/IIIa antibodies) (88). Once these antibodies bind to platelets, the opsonized platelets are recognized and cleared by the mononuclear phagocyte system in the spleen and liver, thereby shortening their circulatory lifespan (89). An alternative hypothesis suggests that coal dust or silica particles can activate macrophages in pneumoconiosis patients (90). These hyperactivated macrophages are capable of not only phagocytosing silica particles but also directly engulfing and sequestering circulating platelets (91, 92). Based on the findings discussed above, the observed thrombocytopenia in pneumoconiosis patients appears to be closely associated with immune dysregulation. Therefore, further investigation is warranted to elucidate the underlying causes and precise molecular mechanisms responsible for platelet reduction in this patient population.

Bilirubin has a variety of biologically active, anti-inflammatory effects, and cellular protective properties against oxidative stress. Lung dust inhalation is associated with lung inflammation and oxidative stress (93, 94). Therefore, bilirubin may be used to reduce oxidative stress after lung dust. Recently, serum bilirubin has been introduced as a potential biomarker in patients with pneumoconiosis (95). Bilirubin, an endogenous lipophilic antioxidant, has well-documented antioxidant properties, and serum bilirubin is considered a potential blood-based indicator of systemic oxidative stress (96, 97). In the early stage of CWP, decreased serum bilirubin levels may reflect elevated reactive ROS in affected individuals, indicating that the body is undergoing oxidative stress (95). A non-targeted metabolomics study in CWP demonstrated reduced bilirubin concentrations in stage I patients, a finding that is consistent with our observations in early-stage cases (95). As the disease advances, patients may progress into a fibrotic stage. Studies in idiopathic pulmonary fibrosis (IPF) have reported reduced heme levels, likely influenced by mitochondrial dysfunction and related metabolic disturbances (98). Further evidence from You-Fan Peng and colleagues shows that direct bilirubin levels are lower in stage III CWP compared with stages I and II, and that serum direct bilirubin is negatively associated with radiological severity. In contrast, indirect bilirubin levels did not differ significantly, suggesting that total serum bilirubin declines in the later stages of the disease (99). The results of this study indicate that the TB in the CP group is significantly lower than that of the control group and suggest that lower bilirubin may have a positive contribution to the occurrence of pneumoconiosis, which may be due to low serum bilirubin levels due to excessive bilirubin consumption in pneumoconiosis patients. A study by You-Fan Peng et al. (99) also demonstrated that serum direct bilirubin levels are inversely correlated with disease severity in pneumoconiosis patients. Therefore, lower levels of bilirubin may suggest the development of pneumoconiosis.

The SHAP analysis in this study indicated that ALT and AST had relatively minor influences on the development of pneumoconiosis. ALT and AST are blood biochemical markers used to assess liver function. In one report (100), elevated AST and ALT were identified as independent predictors of pneumonia (*p* = 0.32) but were not considered to substantially impact pulmonary inflammation. An animal study also showed increased serum levels of AST and ALT in mice with pulmonary fibrosis (101). Although these findings appear inconsistent with our results, they may not be directly generalizable to large-scale clinical studies. As illustrated in Figures 6c,d depicting the individualized probability of pneumoconiosis progression, the SHAP values for ALT and AST were notably lower than those of the other six risk indicators. Overall, current evidence suggests that ALT and AST do not significantly drive the progression of pneumoconiosis. Moreover, reports on the effect of pneumoconiosis on liver function remain relatively limited. Therefore, we intend to conduct further research to investigate whether alanine aminotransferase influences the onset of pneumoconiosis.

ML-based computer-aided detection represents an emerging research domain that has exhibited substantial performance enhancements compared to conventional ML methods. By integrating multi-source data, these algorithms have demonstrated feasibility and effectiveness in improving early diagnostic accuracy (15). This study developed a clinically applicable prediction model for pneumoconiosis using ML algorithms, leveraging a large dataset of accessible and cost-effective blood biomarkers (17). After screening nine prediction models, SVM was considered the optimal model, achieving an AUC of 0.9745 in the training set, 0.9750 in the test set. And SVM showed good performance in accuracy, sensitivity, specificity, PPV, NPV and F1. By analyzing the DCA, calibration curve and PR curve of the model, SVM showed a good clinical applicability evaluation. And in the subsequent learning curve, it can be seen that SVM is not overfitted. In the comparison of multiple models, LightGBM, Random Forest, and AdaBoost showed clear signs of overfitting, as evidenced by their perfect training AUC values (AUC = 1.0000) in Table 4. This pattern is consistent with well-recognized characteristics of ensemble tree methods, which tend to overfit when the feature space is relatively small and the sample size is limited. Importantly, these models were neither used for feature selection nor considered in the construction of the final predictive model. Instead, they served solely as components within a broader comparative framework designed to evaluate multiple algorithms. The purpose of this comparative analysis was to ensure that the final model selection was grounded in objective and transparent performance assessment, incorporating both discrimination metrics and clinical decision-curve analysis. In this context, LightGBM, Random Forest, and AdaBoost demonstrated inferior clinical utility compared with the SVM model ultimately chosen for this study. This shows that SVM can be used well in the early prevention of pneumoconiosis patients in clinical practice. There have been many studies using ML algorithms to diagnose and predict the occurrence of pneumoconiosis. Xiaobing Li (21) combined CT radiomics with clinical features to predict the progression of pulmonary interstitial fibrosis in patients with CP. The results showed that the AUC of clinical, radiomic, and joint models was 0.835, 0.879, and 0. 945. In the test cohort, these models had AUC values of 0.732, 0.750, and 0.845, respectively. The prediction model in this study reaches more than 0.975 in both the training set and the test set, indicating that our model performance is relatively good. Hantian Dong et al. (24) analyzed lung function indicators based on ML algorithms to play an important role in the diagnosis of early CP. The SVM algorithm was proved to be the optimal ML model for predicting CP, and the AUC values of the ROC curves obtained from the three feature selection methods using the SVM algorithm were 97.78, 93.7 and 95.56%, respectively. The results of Jiaqi Jia et al. (102) showed that the RF-Adboost model had the best prediction accuracy for CP, with an F1 score of 0.8757, which could effectively predict the stage of CP. Both studies used small sample sizes and therefore would not be very convincing in such a large pneumoconiosis population. Viviana Hanampa et al. (103) used ML to diagnose the staging of pneumoconiosis and showed that the XGBoost model outperformed other models in this configuration, achieving an impressive 98% accuracy, 90% accuracy, and 84% F1. Our study also selected SVM as the optimal model, but the clinical applicability of the model was not evaluated, so whether it is applied to clinical practice should be further confirmed. We believe that our study may be better used for early screening and diagnosis of coal workers’ pneumoconiosis. This study did not include patients with multiple complications (including lung cancer, chronic pulmonary heart disease, respiratory failure, spontaneous pneumothorax, tuberculosis and other infections and pulmonary hypertension), who are likely to progress to advanced pneumoconiosis and have no more reference value for early pneumoconiosis diagnosis. This study did not collect imaging data and pulmonary function data of patients, because blood routine indicators are easier to obtain and can greatly reduce patient costs and medical costs. It can also allow more workers exposed to coal dust to speculate whether they have pneumoconiosis through the blood indicators of routine physical examinations every year. Therefore, our research can help frontline clinicians and patients to screen, identify and intervene in the occurrence of pneumoconiosis early, and avoid the rapid progression of pneumoconiosis.

SVM were selected as one of the primary algorithms for disease prediction because of several methodological advantages (104). In our study, SVMs demonstrated the best performance on the clinical decision curve in the test set, indicating their potential utility for large-scale clinical prediction tasks. SVMs are well suited to high-dimensional data and can model complex nonlinear relationships between predictors and clinical outcomes through kernel-based learning (105). The use of a radial basis function (RBF) kernel enabled the classifier to capture intricate interactions among hematologic indicators without requiring manual specification of higher-order terms (105). In addition, the soft-margin formulation provides tolerance to moderate measurement noise inherent in biological assays, while probability estimates obtained via Platt scaling facilitate individualized risk prediction within clinical decision-support workflows (106). In our analysis, the SVM model performed well in generating individualized estimates, allowing the evaluation of how each selected risk factor contributes to the development of CWP. Despite these strengths, several limitations associated with SVMs and their practical implementation should be acknowledged. First, SVM performance is sensitive to hyperparameters such as the regularization parameter (C) and kernel coefficient (*γ*); suboptimal tuning may lead to underfitting or overfitting (107). To reduce this risk, hyperparameter selection was informed by ten-fold cross-validation, which helped stabilize model performance and limit overfitting—consistent with the concordant results observed between the training and test sets. Second, SVMs require careful scaling of input variables (108). To minimize methodological bias, missing numerical values were imputed appropriately, and extreme observations were handled using standardized outlier-management criteria to prevent distortion of the feature space. Finally, we recognize that SVMs offer limited intrinsic interpretability (109). To enhance transparency and reduce dimensionality before model fitting, we applied LASSO regression for feature selection and subsequently confirmed the statistical relevance of retained predictors using multivariable logistic regression. This two-stage approach ensured that only clinically meaningful variables were incorporated into the final SVM model. We observed clear overfitting in LightGBM, Random Forest, and AdaBoost on the training dataset. To address the reviewer’s concern, we examined whether any hematological variables might serve as proxies for diagnostic labels. Among the included features, white blood cell count, neutrophils, and lymphocytes represent inflammatory markers that reflect secondary physiological responses related to pneumoconiosis—such as pulmonary inflammation—rather than elements of the diagnostic process itself. Previous studies have shown that indicators such as D-dimer (DD), albumin/globulin ratio (A/G), lactate dehydrogenase (LDH), and white blood cell count (WBC) can contribute to logistic regression models for pneumoconiosis detection; however, these routine blood indices function only as auxiliary biomarkers and cannot independently establish a diagnosis (25). Although inhalation of silica or coal dust triggers inflammatory responses in the lung, the presence of elevated inflammatory markers does not equate to a pneumoconiosis diagnosis. These indices also fluctuate under many unrelated inflammatory conditions (e.g., upper respiratory infections, tonsillitis). Therefore, they cannot independently confirm disease status and do not function as “diagnostic surrogates” or label-carrying features. To ensure transparency, we conducted an additional screening analysis and confirmed that none of the variables were directly related to the radiographic or occupational criteria that determine case classification. We therefore found no evidence of label leakage. To further investigate the source of the perfect AUC values in the training data, we carefully reviewed the training scripts. The near-perfect performance observed in these models is attributable to their inherently high-capacity default configurations rather than to any leakage of diagnostic information. Because our study did not perform hyperparameter optimization and relied entirely on fixed default parameters, this likely contributed to the overfitting as well. Importantly, none of the three models achieved perfect or near-perfect performance on the test dataset (AUC 0.9641–0.9767), indicating that the predictors provide genuine discriminatory information. This further supports the absence of data leakage and demonstrates that, although these models are expressive, they did not encode diagnostic labels through any specific predictor.

The primary aim of our study was to facilitate the early prediction of CWP. Because our research population consisted exclusively of individuals exposed to coal dust and those without existing pneumoconiosis, we developed a simplified online prediction tool (https://sxu25.shinyapps.io/coal-dust-prediction/) based on this cohort. The model is intended for use among workers employed long-term in coal mining or other occupations involving prolonged coal-dust exposure. It is not designed for individuals exposed primarily to silica, iron ore, or other types of industrial dust. Our findings are not applicable to female coal dust-exposed populations, as this study did not include any female participants. After routine occupational health examinations conducted at designated occupational-disease hospitals or tertiary medical centers, these coal dust–exposed workers can use our model to estimate their individual risk of developing CWP. The model requires only six routinely measured laboratory indicators—WBC, ANC, PDW, ALT, AST, and TB—from the annual health report to generate a probability of disease. This directly serves our research objective: enabling the assessment of CWP risk without reliance on chest radiography or other imaging tests, thereby substantially reducing diagnostic costs. The model is straightforward to use, requiring only the input of standard laboratory values. While radiological findings remain the gold standard for diagnosing CWP, our tool is intended solely as an adjunct to assist clinicians. Physicians may review the predicted probability and recommend further imaging examinations for individuals with elevated risk, whereas those with lower predicted probabilities may be monitored longitudinally through annual hematologic assessments, reducing unnecessary medical expenditures. In our coal dust–exposed cohort, the highest disease probability estimated by the SVM model was 0.7345. This relatively high value suggests that individuals exceeding this threshold may warrant additional radiological evaluation. Nonetheless, this threshold is intended as a reference point only; it should not replace clinical judgment, nor should it independently guide diagnostic decisions, in order to avoid misclassification or delayed detection of disease progression.

However, our study has several limitations. First, although 935 patients were included in this study, the sample size in this study was still relatively small; And the data are a study in one center in one region, not a multicenter study. Therefore, the generalizability of the results is limited. Secondly, there is no information on the duration and intensity of dust exposure, smoking status, and past respiratory diseases in this study, which poses a challenge to the comprehensive analysis of CP as the physical examination center does not have clinical data for these patients. In future research, we plan to systematically collect information on these potential indicators to analyze the correlation between these factors and key hematological variables, and further evaluate whether these factors may affect the predictive performance of the model. In addition, this data does not track changes in these metrics for each patient, leading to a decrease in prediction accuracy and overlooking disease progression. In subsequent studies, we will further investigate the dynamic patterns and underlying mechanisms of these 6 risk factors across different stages of coal workers’ pneumoconiosis.

## 5 Conclusion

In conclusion, 6 predictors related to the occurrence of pneumoconiosis were screened out by using the blood indexes of coal miners’ routine physical examination, namely WBC, PDW, TB, ANC, ALT and AST. The 6 features synthesis was substituted into the ML model to construct a prediction model, and the SVM model showed good performance and clinical applicability in this study. In addition, we analyzed the contribution of each influencing factor to the occurrence of pneumoconiosis through SHAP, and also provided personalized risk assessment for pneumoconiosis patients. Our research results confirm that the SVM prediction model has great application prospects in clinical practice, which can help doctors better screen and diagnose pneumoconiosis patients at a better time. Future work will focus on further validating the model’s effectiveness and exploring the integration of these predictive tools into clinical practice.

## Data Availability

The raw data supporting the conclusions of this article will be made available by the authors, without undue reservation.

## Glossary

ML: Machine learning
LASSO: Least Absolute Contraction and Choice Operator
LR: Logistic Regression
XGBoost: EXtreme Gradient Boosting
LightGBM: Light Gradient Boosting Machine
RF: Random Forest
AdBoost: Adaptive Boosting
GNB: Gaussian Naïve Bayes
MLP: Multilayer Perceptron
SVM: Support Vector Machine
KNN: K-Nearest Neighbors
PPV: Positive predictive value
NPV: Negative predictive value
DCA: Decision curve analysis
PR: Precision-recall
SHAP: Shapley Additive exPlanations
ROC: Receiver operating characteristic curve
RBC: Red Blood Cell
PL: Platelet
HB: Hemoglobin
ANC: Absolute Neutrophil Count
NP: Neutrophil Percentage
ALC: Absolute Lymphocyte Count
LP: Lymphocyte Percentage
AEC: Absolute Eosinophil Count
EP: Eosinophil Percentage
ABC: Absolute Basophil Count
BP: Basophil Percentage
PDW: Platelet Distribution Width
ALT: Alanine Aminotransferase
AST: Aspartate Aminotransferase
WBC: White Blood Cell
MPV: Mean Platelet Volume
TC: Total Cholesterol
TR: Triglycerides
HDL-C: High Density Lipoprotein Cholesterol
LDL-C: Low Density Lipoprotein Cholesterol
TB: Total Bilirubin
